# Genomic identification and functional analysis of essential genes in *Caenorhabditis elegans*

**DOI:** 10.1186/s12864-018-5251-3

**Published:** 2018-12-04

**Authors:** Shicheng Yu, Chaoran Zheng, Fan Zhou, David L. Baillie, Ann M. Rose, Zixin Deng, Jeffrey Shih-Chieh Chu

**Affiliations:** 1 Key Laboratory of Combinatorial Biosynthesis and Drug Discovery, Ministry of Education, School of Pharmaceutical Sciences, Wuhan University, Wuhan, 430071 China; 2 Wuhan Frasergen Bioinformatics, Wuhan East Lake High-tech Zone, Wuhan, 430075 China; 3 Department of Molecular Biology and Biochemistry, Simon Fraser University, Burnaby, BC V5A 1S6 Canada; 4 Department of Medical Genetics, University of British Columbia, Vancouver, BC V6T 1Z4 Canada

**Keywords:** Essential gene, Lethal, Genetic balancer, Whole genome sequencing (WGS), Functional characterization

## Abstract

**Background:**

Essential genes are required for an organism’s viability and their functions can vary greatly, spreading across many pathways. Due to the importance of essential genes, large scale efforts have been undertaken to identify the complete set of essential genes and to understand their function. Studies of genome architecture and organization have found that genes are not randomly disturbed in the genome.

**Results:**

Using combined genetic mapping, Illumina sequencing, and bioinformatics analyses, we successfully identified 44 essential genes with 130 lethal mutations in genomic regions of *C. elegans* of around 7.3 Mb from Chromosome I (left). Of the 44 essential genes, six of which were genes not characterized previously by mutant alleles, *let-633/let-638* (B0261.1), *let-128* (C53H9.2), *let-511* (W09C3.4), *let-162* (Y47G6A.18), *let-510* (Y47G6A.19), and *let-131* (Y71G12B.6). Examine essential genes with Hi-C data shows that essential genes tend to cluster within TAD units rather near TAD boundaries. We have also shown that essential genes in the left half of chromosome I in *C. elegans* function in enzyme and nucleic acid binding activities during fundamental processes, such as DNA replication, transcription, and translation. From protein-protein interaction networks, essential genes exhibit more protein connectivity than non-essential genes in the genome. Also, many of the essential genes show strong expression in embryos or early larvae stages, indicating that they are important to early development.

**Conclusions:**

Our results confirmed that this work provided a more comprehensive picture of the essential gene and their functional characterization. These genetic resources will offer important tools for further heath and disease research.

**Electronic supplementary material:**

The online version of this article (10.1186/s12864-018-5251-3) contains supplementary material, which is available to authorized users.

## Background

Essential genes are absolutely required for the viability of an organism such that loss of function mutation in essential genes will lead to lethality or unviable progeny [1, 2]. Recent research has shown that essential genes are associated with human diseases and conditions such as miscarriages [3, 4] and cancers [5–7]. The discovery of many important essential genes, such as *let-60/Ras* [8] and *let-740/dcr-1* [9, 10], were attributed to the use of model organism *Caenorhabditis elegans*, in which essential genes is estimated to take up 25% of all the genes [11–13]. In mammals, approximately one-third of all mammalian genes are essential for life [14].

Due to the importance of essential genes, large scale efforts have been undertaken to identify the complete set of essential genes and to understand their function. For instance, 3326 murine genes were identified to be essential upon knockout, which accounts for 14% of the murine genome [14, 15]. Many of the essential genes in mice are enriched in human disease genes [7, 15], such as cardiovascular (GATA4), neoplasms (KLF6), and nervous system (HOXA1). Similar large-scale loss-of-function studies is also available for several other model organisms including *Saccharomyces cerevisiae* [16, 17], *Schizosaccharomyces pombe* [18], *Drosophila melanogaster* [19–24], and *Danio rerio* [25, 26]. In *C. elegans*, RNAi knock-down phenotypes were examined for roughly 92% of the *C. elegans* genes and about 3500 genes (~ 17%) have been annotated as essential [13, 27, 28].

While RNAi was successful in applying genome-wide targeted approach to identify genetic phenotypes, it is limited to only knock-down gene expression instead of fully knock-out gene expression and are unable to maintain the phenotype over longer periods of time [13, 29]. The best approach is by mutagenesis and screen for gene knock-outs. The concerted effort in the *C. elegans* Deletion Mutant Consortium along with the Million Mutation Project has generated loss-of-function alleles in 13,760 of 20,514 protein-coding genes [30]. The great majority of the mutants from the above resources, however, are largely non-lethal mutations as their approach requires the mutant strain to propagate [30]. An effectively way to screen and maintain lethal mutations is to use genetic balancer systems [31]. Nearly 70% of the *C. elegans* genome is balanced by genomic rearrangements such as duplications, translocations, and inversions [31, 32]. Duplication balancers do not cross-over with normal chromosomes and thereby providing a third allele that carries the wildtype rescuing allele [31]. The large chromosomal duplications are not replicated and they segregate in a non-Mendelian fashion such that it is not pass down to daughter cells equally in meiosis. The progeny inheriting the duplication will survive while the progeny without the duplication will not. Previous genetic studies have identified 103 essential genes mapped to 5.4 Mb region of Chromosome I balanced by the duplication *sDp2* [33]. We have previously combined the mapping data with next generation sequencing to identify the molecular identities of many essential genes but many more are still uncharacterized [27].

Many studies have suggested that genes are not randomly disturbed in the genome. For instance, the chromosomal clustering of housekeeping genes [34] and the distribution biases of the sex-regulated genes [35] can be found in the genome. Recent technological advances in chromatin-conformation capture methods have allowed in-depth study of genome organization. Methods such as 3C [36], 4C [37], Hi-C [38], and ChiA-PET [39, 40] examines genomic fragments that are close in proximity in nuclear space and have been successfully applied to bacteria [41–43], yeast [44–46], *Plasmodium falciparum* [47], plants [48, 49], *C. elegans* [50, 51], fruit fly [52, 53], mouse [54, 55], and humans [38, 55–57]. By crosslinking genomic fragments that are close in space followed by high-throughput sequencing, Hi-C is able to identify the loci that are close in space but not necessarily close in genomic coordinates [38, 57–59]. The chromatin interactions in the genome can form domains called topologically associating domains, or TADs, which are megabase-pair size regions where intra-chromatin interactions occur more frequently than other chromatin regions [55, 60]. TADs share a high degree of similarity in the domain organization across different cell types and are conserved between mice and humans, suggesting that TADs are the stable domain organization in mammalian genomes [55].

Functionally related genes showed higher clustering on the chromosomes [61] and may be linked in their gene expression regulation. Functionally linked genes, including co-expressed genes, genes in common pathway, or genes with protein-protein interaction exhibit higher clustering on chromosomes in both *Escherichia coli* and humans [62, 63]. TAD boundaries, defined as genomic region between TADs, are abundant in transcription start sites, active transcription, active chromatin marks, housekeeping genes, and tRNA genes [55]. These findings inspired us to consider whether genes with same essentiality or co-expression genes have some spatial localization features and whether essential genes show enrichment in TAD boundaries.

## Results

### Identification of genomic mutations in 130 chromosome I mutants

Genomic DNA libraries of 130 mutant strains (Additional file 1) with *dpy-5 (e61)* and *unc-13 (e450)* balanced by *sDp2* were prepared and sequenced using Illumina HiSeq to generate 100 bp paired end reads. We achieved an average sequencing depth of 23X across the whole genome and an average depth of 28X in coding regions. The *dpy-5 (e61)* and *unc-13* (*e450*) identified previously are used as a quality check [27]. For *unc-13*, the variant ratio is expected to be 100% because the *sDp2* does not balance that allele. For *dpy-5*, a 66% variant ratio is expected because the *sDp2* carry a rescuing allele [27]. In our sequencing data, we found 23 strains without the expected *dpy-5 (e61)I* and *unc-13 (e450)I* mutation and they were removed from further analysis. In the case of 4 strains where there is insufficient sequencing (below 8X coverage), *let-394 (h235)*, *let-545 (h842)*, *let-395 (h271)*, and *let-122 (h226)* were also removed from subsequent analyses. As a result, a total of 103 strains were analyzed.

### Identification of essential genes

Improving upon a method previously adapted for identifying lethal mutations on Chromosome I balanced by *sDp2* [27], we identified 58 putative lethal mutations in 103 strains. These putative lethal mutations fall into 44 genes. The full list of let genes with its identified sequences are shown in Table 1 and Additional file 2.

**Table 1:** Biological functions of the identified 44 essential genes

*let-name*	*Essential gene*	*Alleles*	*Allele mutation (nucleic acid)*	*Allele mutation (Protein)*	*Pfam*	*KOG*	*Evolutionary conservatior*
*let-609*	*let-363*	h191	C- > T	R- > X	Phosphatidylinositol 3- and 4-kinase	Replication, recombination and repair	I,F,M,N
*let-643*	*nath-10*	h500	G- > A	R- > K	GNAT acetyltransferase 2	General function prediction only	I,F,M,N
*let-624/ let-644/ let-622*	*npp-6*	h449,h839,h222	C- > T	Q- > X	Nucleoporin Nup120/160	Unkown	I,F,M,N
*let-610*	*asd-2*	h695	C- > T	P- > S	Homodimerisation region of STAR domain protein	RNA processing and modification	I,F,M,N
*let-639/ let-371*	*hcp-6*	h779,h123	G- > A	W- > X	non-SMC mitotic condensation complex subunit 1	Function unknown	I,F,M,N
*let-138/ let-150/ let-357*	*spg-7*	h744,h282,h89	G- > A	W- > X	Peptidase family M41	Posttranslational modification, protein turnover, chaperones	I,F,M,N
*let-163*	*sep-1*	h483	C- > T	Q- > X	Peptidase family C50	Cell cycle control, cell division. Chromosome partitioning	F,M,N
*let-133*	*Y71G12B.8*	h440	T- > A	Y- > X	DEAD/DEAH box helicase	RNA processing and modification	I,F,M,N
*let-593*	*inx-13*	h212	C- > T	Q- > X	Innexin	Unkown	I,F,N
*let-625*	*rpl-4*	h506	G- > A	D- > N	Ribosomal protein L4/L1 family	RNA processing and modification	I,F,M,N
*let-633/ let-638*	*B0261.1*	h696,h778	C- > T	R- > X	Myb DNA-binding like	Transcription	I,F,M,N
*let-648*	*vha-16*	h781	G- > A	D- > N	Unkown	Unkown	I,N,M,F
*let-615*	*rpl-13*	h529	C- > T	Q- > X	Ribosomal protein L13e	Translation, ribosomal structure and biogenesis	I,F,M,N
*let-356*	*cdc-6*	h501	G- > A	G- > R	ATPase family associated with various cellular activities (AAA)	Cell cycle control, cell division. Chromosome partitioning;Replication, recombination and repair	I,F,M,N
*let-505*	*tufm-2*	h426	C- > T	R- > X	Elongation factor Tu GTP binding domain	Translation, ribosomal structure and biogenesis	I,F,M,N
*let-128*	*C53H9.2*	h253	465 + 1G > A	None	50S ribosome-binding GTPase	General function prediction only	I,F,M,N
*let-398*	*gpc-2*	h257	C- > T	Q- > X	GGL domain	Signal transduction mechanisms	I,F,M,N
*let-619/ let-105*	*dip-2*	h348,h681	C- > T	H- > Y	AMP-binding enzyme	General function prediction only	I,F,M,N
*let-649/ let-109*	*him-1*	h491,h811	G- > A	G- > R	RecF/RecN/SMC N terminal domain	Cell cycle control, cell division. Chromosome partitioning	I,N,M,F
*let-578*	*npp-11*	h512	G- > A	W- > X	Nucleoporin FG repeat region	Intracellular trafficking, secretion, and vesicular transport;Nuclear structure	I,F,M,N
*let-543/ let-544*	*sacy-1*	h792,h692	G- > A	G- > R	DEAD/DEAH box helicase	RNA processing and modification	I,F,M,N
*let-614*	*rmh-1*	h147	C- > T	S- > L	RecQ mediated genome instability protein	Function unknown	F,N
*let-582*	*egg-4*	h726	G- > A	A- > T	Protein-tyrosine phosphatase	Signal transduction mechanisms	I,F,M,N
*let-528*	*cytb-5.2*	h1012	G- > A	W- > X	Cytochrome b5-like Heme/Steroid binding domain	Energy production and conversion	I,F,M,N
*let-511*	*W09C3.4*	h755	262-1G > A	None	RNA polymerase Rpc34 subunit	Transcription	I,F,M,N
*let-135*	*pop-1*	h268	G- > A	A- > T	HMG (high mobility group) box	Transcription	I,F,M,N
*let-502*	*spe-5*	h767	C- > T	S- > F	ATP synthase alpha/beta family, nucleotide-binding domain	Energy production and conversion	I,F,M,N
*let-143*	*npp-13*	h513	G- > A	G- > E	Nup93/Nic96	Cell cycle control, cell division. Chromosome partitioning	I,F,M,N
*let-571*	*eif-2gamma*	h347	G- > A	G- > R	Initiation factor eIF2 gamma, C terminal	Translation, ribosomal structure and biogenesis	I,F,M,N
*let-155*	*inx-21*	h461	C- > T	R- > X	Innexin	Unkown	I,F,N
*let-162*	*Y47G6A.18*	h460	G- > A	G- > E	Golgi phosphoprotein 3 (GPP34)	Intracellular trafficking, secretion, and vesicular transport	I,F,M,N
*let-510*	*Y47G6A.19*	h740	1355-1G > A	None	Zinc carboxypeptidase	Function unknown	I,F,M,N
*let-357*	*lpd-3*	h132	1539 + 1G > A	None	Fragile site-associated protein C-terminus	Unkown	I,F,M,N
*let-546*	*xpo-2*	h227	G- > A	W- > X	Cse1	Intracellular trafficking, secretion, and vesicular transport;Nuclear structure	I,F,M,N
*let-121/ let-146*	*cdt-1*	h810,h197	C- > T	Q- > X	DNA replication factor CDT1 like	Unkown	I,F,M,N
*let-130*	*lpr-1*	h773	G- > A	R- > Q	Unkown	Unkown	F,N
*let-573*	*rpl-1*	h247	C- > T	T- > I	Ribosomal protein L1p/L10e family	Translation, ribosomal structure and biogenesis	I,F,M,N
*let-145*	*arx-1*	h182	C- > T	Q- > X	Actin	Cytoskeleton	I,F,M,N
*let-123/ let-142/ let-583*	*cogc-3*	h413,h518,h738	G- > A	G- > R	Sec34-like family	Intracellular trafficking, secretion, and vesicular transport	I,F,M,N
*let-577*	*sop-3*	h503	G- > A	E- > K	Unkown	Unkown	F,M,N
*let-548/ let-144*	*tln-1*	h356,h393	C- > T	Q- > X	Talin, middle domain	Cytoskeleton	I,F,M,N
*let-131*	*Y71G12B.6*	h817	C- > T	Q- > X	GDP-mannose 4,6 dehydratase	Unkown	F,N
*let-392*	*nekl-2*	h120,h122	G- > A	G- > E	Protein kinase domain	General function prediction only	F,M,N
*let-374*	*lpd-5*	h251	G- > A	W- > X	Unkown	Unkown	I,F,M,N

Table includes 44 identified essential genes in this study with the information of *let-x* name, Alleles, Allele mutation, biological functions, and evolutionary conservation. N-Nematodes, I-Invertebrates (*Drosophila*), M-Mammals (mouse, human), F-Fungi (*Saccharomycetaceae*)

### Novel essential genes identified

Of the essential genes we have identified, we found 6 new putative essential genes in which no other knock-out alleles have been generated. Of these 6 genes, *let-633/let-638* (B0261.1) is orthologous to a novel Myb-like leucine zipper transcription factor, which is necessary for cell proliferation, apoptosis, and differentiation, and plays an important role in the pathogenesis of adenoid cystic carcinoma [64–66]. *let-128* (C53H9.2) is orthologous to 50S ribosome-binding GTPase, as previously research show many *Escherichia coli* GTPases are important in ribosome biogenesis [67]. Mitomycin C induced mutations in this gene also shows this gene as essential for survival [68]. *let-511* (W09C3.4) is orthologous to RNA polymerase Rpc34 subunit, which plays a key role in the recruitment of RNAP III to the pre-initiation complex [69, 70]. *let-162* (Y47G6A.18) is orthologous to the Golgi phosphoprotein 3, which is a peripheral membrane protein of the Golgi stack and plays a regulatory role in Golgi trafficking [71]. *let-510* (Y47G6A.19) is orthologous to zinc carboxypeptidase, which plays a role of protease enzyme that hydrolyzes peptide bonds at the carboxy-terminal end of a protein or peptide. *Let-131* (Y71G12B.6) is orthologous to GDP-mannose 4,6 dehydratase, which is essential in the first step of GDP-fucose biogenesis pathway [72].

### Functions of the identified 44 essential genes

To understand the biological roles of essential genes, we first examined the functions of the 44 essential genes identified in this study based on their orthologous genes (Table 1). Among the 44 genes, 13 essential genes encode enzymes, such as 50S ribosome-binding GTPase, RNA polymerase Rpc34 subunit, ATP synthase alpha/beta family, protein-tyrosine phosphatase, and nucleotide-binding domain. We found 5 genes related to ribosome biology and biogenesis (Additional file 3. column: KEGG). Twelve essential genes were found to be involved in protein metabolic processes (Additional file 3).

Considering that the biological roles of essential genes is very important, essential genes are often conserved across different species. We investigated the orthologs of these essential genes in other nematodes (N), Invertebrate (I) (*D. melanogaster*), Mammals (M) (mouse and human), and Fungi (F) (of the family *Saccharomycetaceae*) as shown in Table 1. We found that 35 of 44 (79.5%) essential genes were conserved in all the examined organisms. Three of the genes were found to be essential in fungi and nematodes, such as *let-30/lpr-1*, a required gene at a time of rapid luminal growth expressed by the duct, pore and surrounding cells [73]. Three genes were found in nematodes, fungi, and mammals, such as, *let-163/sep-1* is a member of peptidase family C50*,* encodes the *C. elegans* ortholog of separase, a cysteine protease first discovered in yeast, *sep-1* activity is required for a number of cell cycle events including sister chromatid separation and membrane trafficking [28]. We found two genes specific to invertebrates, which were conserved in nematodes, fungi, and invertebrates, but not in mammals. For instance, *let-593/inx-13* encodes an innexin, which is an essential transmembrane channel protein and involved in the building of invertebrate gap junctions.

### Gene essentiality analysis

**Fig. 1:**
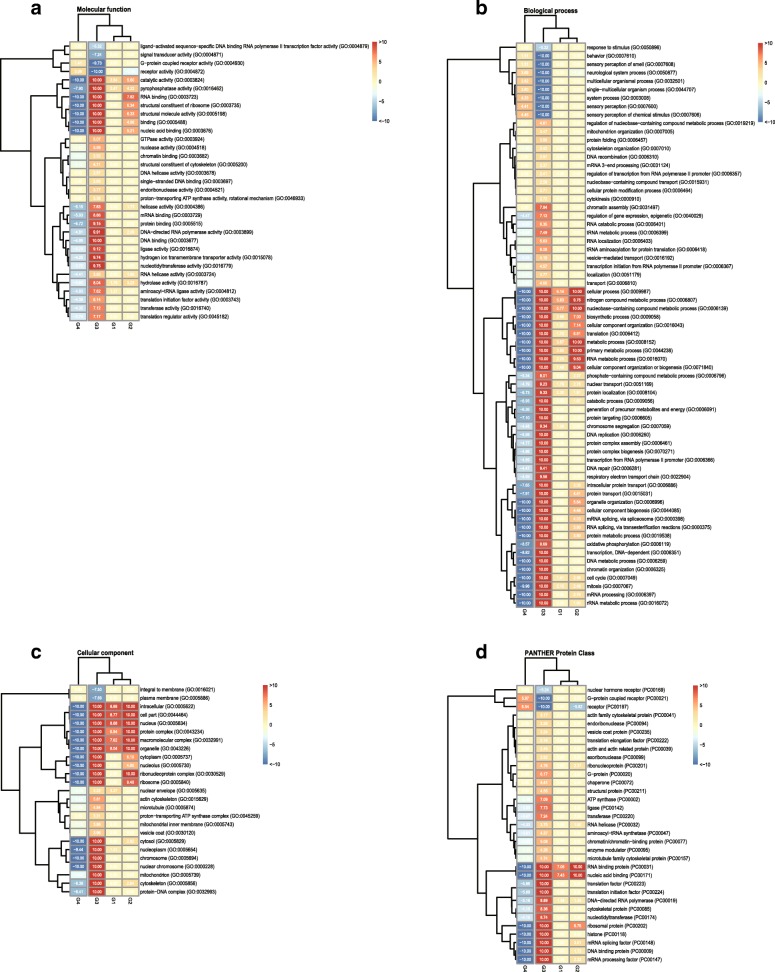
The heat map analysis for the significant conserved gene functions based on PANTHER Overrepresentation Test. The hierarchal cluster diagram was constructed by using pheatmap clustering in R. The *P*-values of each annotation data set (**a**) Molecular Function, (**b**) Biological Process, (**c**) Cellular Component, and (**d**) PANTHER Protein Class) are calculated with the Bonferroni correction for multiple testing by each functional group, which reflect the significance of the difference for the enrichment value between the essential and nonessential genes. The red boxes represent that the functional group are overrepresentation, while the blue boxes represent the opposite case. Conversion *P*-value to -log10 (x), and get the heatmap of the converted *p*-value, the conversion value is thought to be 0 while the *P*-value is greater than 0.05, the conversion value is thought to be 10 while the *P*-value is less than 0.0000000001

To conduct gene essentiality analysis, four groups of genes were used for comparison: Group one (G1): essential genes that were isolated through genetic screens and are fully sequenced and analysed by high throughput methods dependent on the use of allelic ratios [27, 33, 74] (82 in total). Group two (G2): essential genes that have published alleles or RNAi supporting lethal phenotypes in the region of chromosome I balanced by *sDp2* (366 in total). Group three (G3): essential genes that have published alleles or RNAi supporting lethal phenotypes (3083 in total). Group four (G4): non-essential genes that have no observable lethal phenotypes caused by either RNAi or known alleles (16,018 in total). We compared the function of essential genes from four groups based on GO annotations (Cellular Component, Biological Process, and Molecular Function) and PANTHER Protein Classification (Fig. 1).

For the Molecular Function annotation analysis, genes from G1, G2, and G3 do not show significant difference in any Molecular Function annotation. However annotations such as catalytic activity (GO:0003824) (*P-value* = *4.77e*^*− 17*^) and pyrophosphatase activity (GO:0016462) (*P-value* = *1.27e*^*− 8*^) are significantly underrepresented in G4 (Fig. 1a). This is consistent with our observation in the cellular component analysis, in which annotations of the intracellular (GO:0005622) (*P-value* = *2.74e*^*− 132*^), protein complex (GO:0043234) (*P-value* = *4.40e*^*− 70*^), and macromolecular complex (GO:0032991) (*P-value* = *6.47e*^*− 129*^) are overrepresented in G3 (Fig. 1b). With regard to the biological processes, essential genes in G3 are significantly enriched for cellular process (GO:0009987) (*P-value* = *6.06e*^*− 99*^), as well as nitrogen compound metabolic process (GO:0006807) (*P-value* = *1.28e*^*− 80*^) and nucleobase−containing compound metabolic process (GO:0006139) (*P-value* = *4.69e*^*− 133*^), suggesting that essential genes tend to be involved in protein synthesis. In contrast, G4 protein products are significantly enriched for the regulation of system process (GO:0003008) (*P-value* = *4.65e*^*− 5*^), such as sensory perception (GO:0007600) (*P-value* = 3.90*e*^*− 5*^), neurological system process (GO:0050877) (*P-value* = 2.06*e*^*− 4*^), and multicellular organismal process (GO:0032501) (*P-value* = 1.52*e*^*− 4*^). If there are disruptions in these processes, *C. elegans* might show mutant phenotypes, which however, are most likely not lethal. According to PANTHER Protein Class analysis, we found that essential genes in G3 are significantly enriched for nucleic acid binding (PC00171) (*P-value* = 3.50*e*^*− 128*^), and RNA binding protein (PC00031) (*P-value* = 9.97*e*^*− 113*^).

All in all, the above analysis suggests that essential genes plays a key role in enzyme and nucleic acid binding activities during fundamental processes, such as DNA replication, transcription, and translation.

### Gene essentiality vs. gene cluster

**Fig. 2:**
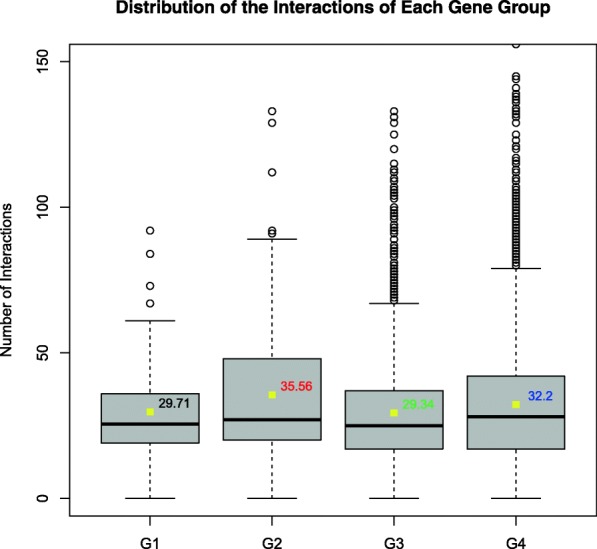
The cluster frequency of different group genes in mixed-stage embryos *C. elegans*. Box plots of each group genes for gene interactions frequency. The numbers on the right side of the yellow block represent the average interactions frequency of genes in each group. The *P*-values were obtained from the Mann-Whitney U test / the Wilcoxon rank sum test after the Levene’s test

It has been noted before that gene essentiality, evolutionary conservation, interaction networks, and gene expression are biological factors that can influence the structural features of proteins [75]. Thus, we decided to assess the properties of essential genes between the 4 groups from three different perspectives: gene cluster, gene expression, and protein connectivity. Hi-C experiments aims to capture the DNA fragments that are close in spatial proximity and genes that are close in space tend to share common functionality [62]. We aim to use Hi-C data to determine whether essential genes exhibit higher or lower gene cluster densities. The contact frequencies between all genes were derived from the Hi-C interacting DNA fragments of Wild-Type (N2) mixed-stage embryos of *C. elegans* [50]. Then, the average contact frequencies of genes in each group were calculated. Figure 2 shows genes from G2 tend to have more interaction partners than other essential/ non-essential genes. We observed that genes from G2 tend to have more interaction partners than G1 (*P-value = 3.08e*^*− 2*^*,* Mann-Whitney U test), which means the essential genes, sequenced and analysed by our high throughput method, tend to have less interaction partners than the other essential genes in the region of chromosome I balanced by *sDp2*. Genes from G2 also have more interaction partners than G3 (*P-value = 1.62e*^*− 4*^*,* Wilcoxon Rank Sum test), which might be due to fact that G2 essential genes are enriched in in cell cycle control, transcriptional regulation, and RNA processing [27]. G2 also have more interaction partners than G4 (*P-value = 1.89e*^*− 2*^*,* Mann-Whitney U test), which indicates essential genes in the region of chromosome I balanced by *sDp2* tend to engage in larger gene cluster than to non-essential genes. However, we do see that G4 tend to have more interaction partners than G3 (*P-value = 6.10e*^*− 8*^*,* Wilcoxon Rank Sum test), suggesting non-essential genes tend to engage in larger gene cluster than to essential genes in general.

### Gene essentiality vs. TAD boundaries and gene expression

**Fig. 3:**
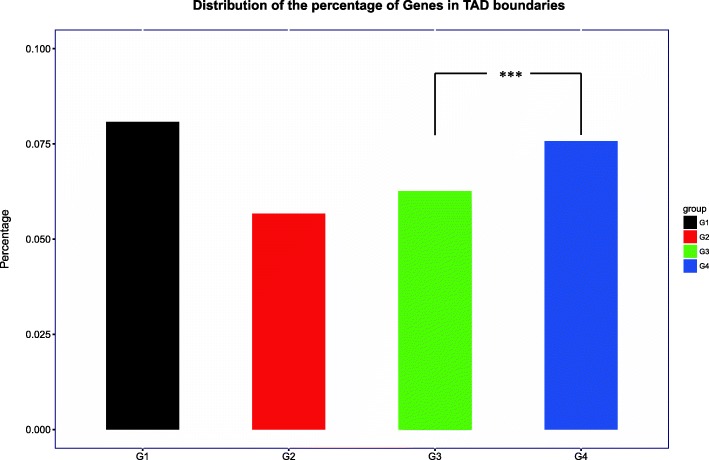
The percentage of different essentiality genes locate in TAD boundaries. The four groups are labelled in black, red, green, and blue, respectively. Statistical difference was calculated for G1 vs. G2, G3, and G4 individually by using Fisher’s exact test (* *P*-value < 0.05, ** *P*-value < 0.01, *** *P*-value < 0.001)

**Fig. 4:**
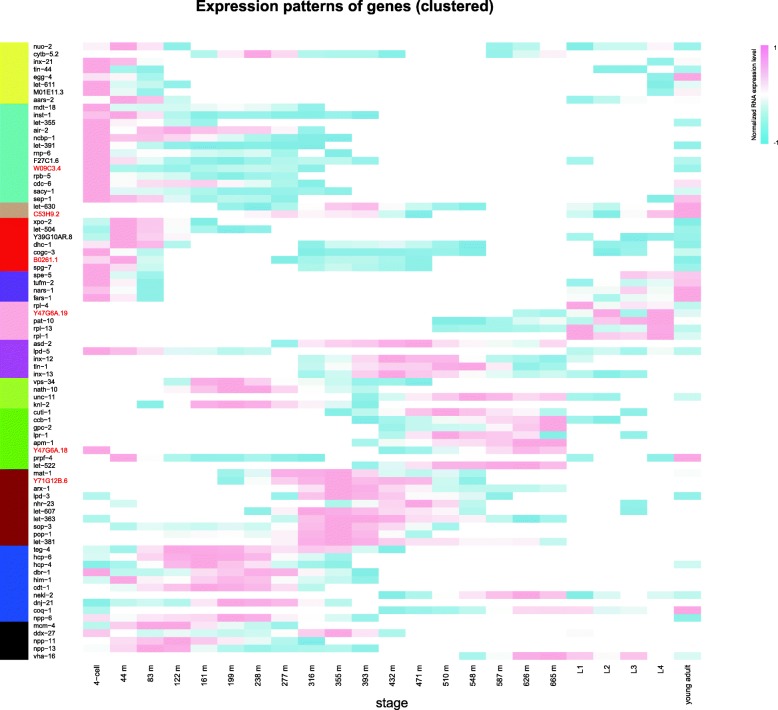
The Gene expression: This figure represents the normalized transcript level (read number per coding length per million reads) for each gene across the developmental stages including 18 embryo stages, four larval stages (L1-L4), and young adult. To facilitate comparison, we subtract each gene expression values in different periods from the average value of the gene expression in different periods. The heatmap represents normalized transcript level from high (pink) to low (skyblue). Eight distinct modules that are based on their expression pattern are shown by colored modules. Yellow, Turquoise, Red, Purple, Blue, and Black: early-embryonic; Magenta: early- and mid-embryonic; Tan and Brown,: mid-embryonic; Green: late-embryonic; Greenyellow: early-, mid- and late-embryonic; Pink: larval

TAD boundaries are enriched in transcription start sites, active transcription, active chromatin marks, housekeeping genes, tRNA genes, short interspersed nuclear elements (SINEs), as well as binding sites for architectural proteins like CTCF and cohesin [55, 76–79]. To test whether essential genes tend to cluster in TAD boundaries, we examined the genes in each group and its association with TADs. Figure 3 shows G4 has higher probability than G3 to be in TAD boundaries (*P-value = 8.33e*^*− 3*^*, Fisher’s exact test*) and seems that more essential gene tend to locate within TAD domains instead of at the boundaries. The fact that essential genes are not enriched in TAD boundaries suggest that essential genes expression may not be constitutively expressed like most house-keeping genes. Indeed, when we examined the gene expression of essential genes using weighted correlation network analysis (WGCNA) over 23 developmental stages, we found that essential genes are expressed in specific time frames with most of the essential genes show strong expression in early development (Fig. 4).

### Gene essentiality vs. protein connectivity

**Fig. 5:**
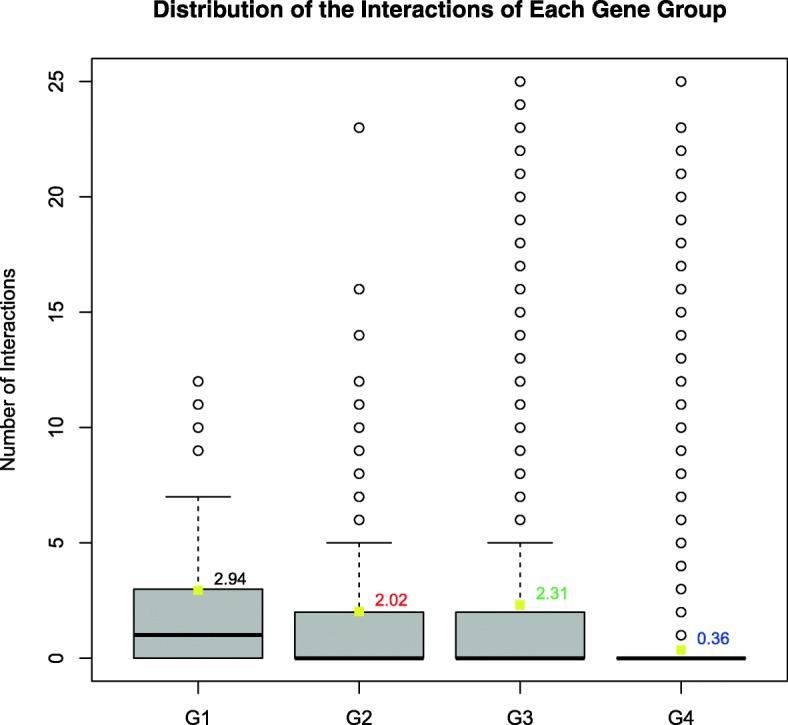
The protein interaction frequency of different group genes. Interaction number distribution of each group with whole genome protein interactions in *C. elegans*. Box plots of each group for protein interaction frequency. The numbers on the right side of the yellow block represent the average interaction frequency of proteins in each group. The *P*-values were obtained from the Mann-Whitney U test / the Wilcoxon rank sum test after the Levene’s test

We hypothesize that essential genes will have more protein-protein interactions than to non-essential genes due to its functional importance. Figure 5 shows the distribution of the number of protein-protein interactions. Proteins from G4 tend to have less interaction partners than G3 (*P-value < 2.20e*^*− 16*^*,* Wilcoxon Rank Sum test), suggesting that essential genes tend to be protein interaction hubs. Similar results are seen for G1 (*P-value < 2.20e*^*− 16*^*,* Wilcoxon Rank Sum test) and G2 (*P-value < 2.20e*^*− 16*^*,* Wilcoxon Rank Sum test) in comparison with G4.

## Discussion

Using genetic mapping, Illumina sequencing, and bioinformatics analyses, we successfully identified 44 essential genes with 130 lethal mutations in genomic regions of *C. elegans* of around 7.3 Mb from Chromosome I (left). From the 44 essential genes we have identified, we found 6 new predicted essential genes. As a result of our study, the total essential genes identified in the region covered by *sDp2* is now 82. High-throughput sequencing of balanced lethal mutations has proved that it is more efficient and cost-effective than the traditional method, which undertakes dozens of Sanger sequencing of genes in a particular genetic mapping zone. Depending on the size of the mapped zone, traditional method can take months or years to characterize one allele.

Essential genes are important for the viability of an organism and can play a key role in novel drug development [1, 2]. With approximately 60% of the essential genes showing human orthologs, *C. elegans* is also an important multi-cellular animal for the study of human disease [27]. While knock-out collection, targeted KO by CRISPR/Cas9 system, and RNAi screens steadily increased genomic coverage to genome scale [13, 31, 80–82], identifying essential genes in an intact multicellular organism are still limited in terms of recovery and maintenance of lethal mutations [27, 33]. Therefore, a resource such as described here for identifying and studying essential genes in model organisms is an important genetic resource for understanding organization and function of essential genes as well as providing a platform for in-depth functional studies.

The functions of essential genes vary greatly and spread across many pathways. GO term analysis and PANTHER Protein Class analysis indicates that essential genes play a key role in enzyme, protein complex, cellular process and nucleic acid binding activities during fundamental processes, such as DNA replication, transcription, and translation. However, non-essential genes are significantly enriched for the regulation of system process, such as sensory perception, neurological system process, and multicellular organismal process. Previous reports have shown that essential genes in the left half of chromosome I in *C. elegans* function in cell cycle control, transcriptional regulation, and RNA processing [33]. Our study here increased the number of essential genes identified in Chromosome I and further strengthens the notion that DNA replication, transcription, and translation are enriched in this set.

We found that non-essential genes form larger gene clusters than essential genes in general. Non-essential genes can experience gene duplication during evolution more often than essential genes resulting in paralogs cluster in the linear genome as well as 3D chromatin architecture [83, 84]. This may explain why non-essential genes form larger gene clusters in general.

The observation that essential genes in left half of Chromosome I form larger gene clusters than non-essential genes is intriguing. Functionally linked genes, including co-expressed genes, protein-protein interaction genes, and genes in the same pathway cluster together in physical proximity in *Escherichia coli*, *C.elegans* and humans [62, 63, 85]. From the gene expression analysis, we observed that majority of the essential genes are expressed early in development. We hypothesize that there is a common expression regulation facilitated by the chromatin 3D structure. This notion is consistent with our observation that essential genes tend to locate within TAD structures rather than at TAD boundaries. Studies in *Caulobacter crescentus* shows that highly expressed genes are enriched in the boundaries of chromosomal interaction domains (CIDs) [41]. In mammalian cells, TAD boundaries are enriched in transcription start sites, active transcription, active chromatin marks, housekeeping genes, tRNA genes, and short interspersed nuclear elements (SINEs) [55]. The observation that essential genes expression in very specific developmental stages suggest that expression of essential genes are tightly regulated rather than constitutive expression. By being within the TAD structure, the expression of genes can be controlled by either facilitating or preventing loop interaction [60].

Proteins do not function alone. We found essential genes act like hubs in protein-protein interaction with higher number of protein interactions than non-essential genes. Consistent with the study in yeast where the most highly connected genes in the cell are the most important ones for an organism’s viability [86].

## Conclusions

In the present work, we comprehensively analyzing genomic mutations in 130 Chromosome I mutants of *C. elegans* with a combination of targeted and forward mutational approaches [27] and successfully identified 44 essential genes with high confidence, of which 6 are new essential genes never characterized by mutant alleles before. This is also the first time that all essential genes identified to-date has been analyzed together with 3D chromosome conformation data where we found that essential genes are more located within a TAD structure rather than TAD boundaries. The data presented here provides the genetic resource for further functional studies of essential genes and more understanding towards the minimal set of genes and pathway for survival.

## Methods

### *C. elegans* strains

The strains used are provided in Additional file 1. The strains were generated by mutagenizing KR235 [*dpy-5 (e61), +, unc-13 (e450)*/*dpy-5 (e61), unc-15 (e73), +*; *sDp2*] growing in nematode growth medium streaked with *E. coli* OP50 [27, 87]. The maintenance of each strain and the isolation of its genomic DNA were performed as previously described [27]. Library preparation and sequencing was performed by the BC Cancer Agency Genome Science Center.

### Mutation identification procedure

The FASTQ reads were aligned to the *C. elegans* reference genome (WS246) using BWA [88]. GATK [89], and SAMtools [90] were used to called for variants [27]. The candidate essential genes on Chromosome I are rescued by a third wild-type allele on *sDp2*, and thus we focused on finding mutations that exhibit the variant frequencies to be around 66%. In our sequencing data, we removed strains without the expected *dpy-5 (e61)I* and *unc-13 (e450)I* mutation and strains without sufficient sequencing coverage from further analysis. Single nucleotide variations (SNVs) that exhibited the variant ratio between 40 and 90% were filtered from the sequencing data. Two filtration steps were performed: First, some variations could come from the starting strain KR235 that was used for mutagenesis. In order to filter the background variations between the starting strain and the *C. elegans* reference genome, we excluded all variations that identified in KR235 [27, 74]. Second, the variations were required to be supported by at least 8 reads with both forward and reverse directions. After the aforementioned two steps of filtration, the remaining SNVs were subjected to subsequent essential gene identification.

The molecular identification of essential genes on Chromosome I (left) is based on three lines of evidence. First, variations in each strain were screened based on previous genetic mapping data [80, 91, 92]. Second, lethal phenotypes, which are supported by RNAi or existing alleles in WormBase, increase the credibility of the mutations (www.wormbase.org). Last, mutations, such as splicing or nonsense, which usually lead to harmful phenotypes, in the million mutation project (MMP) database should be absent in essential genes [30]. Thus, it is less likely that the candidate essential genes in the MMP database contain lethal mutations. With the aforementioned information, in total, 44 sequenced essential genes were identified with high confidence in the Chromosome I balanced regions, 9 of which were found in our previous study [27], which were summarized in Table 1 and Additional file 2.

### Essential genes functional analysis

Pfam analysis: The domain families present in each protein was searched with InterProScan [93] using the Pfam database [94].

Gene Ontology (GO) analysis: GO annotation was done using Blast2GO [95]. This part of the analysis was also done by the PANTHER classification system [96] from the website http://pantherdb.org/. GO annotations (Cellular Component, Biological Process, and Molecular Function) and Protein Class (PANTHER Protein Class, are grouping terms to classify protein families and subfamilies, that are sometimes but not always related to molecular function. [97]) were examined individually. Use the Bonferroni correction for multiple testing.

Gene cluster: The Hi-C and TAD data of Wild-Type (N2) mixed-stage embryos of *C.elegans* were obtained from Crane et al. [50]. The data were binned into 50 kb non-overlapping genomic intervals, which we termed as locus. The interaction data between loci were normalized using standard ICE methods [98]. The significance of the interaction between a pair of loci was calculated using *Fit-Hi-C* [99] with a minimum 15 contact counts and *P* < 0.01. When a locus showed significant interaction with 2 or more other loci, all interacting loci were grouped together. The genes within a group of interacting loci were considered as interacting genes and the interaction frequency of each gene was counted. The average interaction frequencies of genes in each group were compared. The *P*-values were obtained from the Mann-Whitney U test / the Wilcoxon rank sum test after the Levene’s test.

Protein connectivity: The protein interaction data for *C. elegans* were obtained from BioGRID [100–102]. There are 3911 unique genes involved in 8488 non-redundant protein-protein interactions. We counted the number of protein-protein interactions of each gene and the average protein-protein interaction frequencies of genes in each group were compared. The *P*-values were obtained from the Mann-Whitney U test / the Wilcoxon rank sum test after the Levene’s test.

Gene expression: The gene expression data for *C. elegans* were obtained from the *GExplore* (version 1.4) database [103], which contains developmental stages originated from the NHGRI modENCODE project [104, 105]. The expression profile clustering was done using Weighted correlation network analysis (WGCNA), which was used for detecting clusters (modules) of highly correlated/co-expression genes [106].

## Additional files

Additional file 1: List of alleles studied. The alleles used for WGS are listed in the 2nd column. (XLS 44 kb)

Additional file 2: Identifications of essential genes. Including information about the allele name, the strain name, the genetic mapping zones [33], location, predicted gene, allele mutation, RNAi support, alleles support, and MMP support of the essential genes. The asterisk (*) signify a stop codon. (XLS 43 kb)

Additional file 3: The KEGG annotation and the GO annotation. The KEGG annotation of genes are listed in the 3nd column .The GO annotation of genes are listed in the 4nd column. (XLS 57 kb)

## Abbreviations

EMS: *E*thyl *m*ethane *s*ulfonate
GO: *G*ene *o*ntology
KEGG: Kyoto Encyclopedia of Genes and Genomes
KOG: EuKaryotic Orthologous Groups
MMP: *M*illion *m*utation *p*roject
SNVs: *S*ingle *n*ucleotide *v*ariations
WGCNA: Weighted correlation network analysis
WGS: *W*hole *g*enome *s*equencing
